# Upregulated expression of miR-4443 and miR-4488 in drug resistant melanomas promotes migratory and invasive phenotypes through downregulation of intermediate filament nestin

**DOI:** 10.1186/s13046-023-02878-9

**Published:** 2023-11-27

**Authors:** Vittorio Castaldo, Michele Minopoli, Francesca Di Modugno, Andrea Sacconi, Domenico Liguoro, Rachele Frigerio, Arianna Ortolano, Marta Di Martile, Luisa Gesualdi, Gabriele Madonna, Mariaelena Capone, Roberto Cirombella, Angiolina Catizone, Donatella Del Bufalo, Andrea Vecchione, Maria Vincenza Carriero, Paolo Antonio Ascierto, Rita Mancini, Luigi Fattore, Gennaro Ciliberto

**Affiliations:** 1 Department of Anatomy, Histology, Forensic- Medicine and Orthopedics, Sapienza University of Rome, 00161 Rome, Italy; 2 Preclinical Models of Tumor Progression Unit, Istituto Nazionale Tumori IRCCS ‘Fondazione G. Pascale’, 80131 Naples, Italy; 3 Tumor Immunology and Immunotherapy Unit, IRCCS Regina Elena National Cancer Institute, 00144 Rome, Italy; 4 Clinical Trial Center, Biostatistics and Bioinformatics Unit, IRCCS Regina Elena National Cancer Institute, 00144 Rome, Italy; 5 Department of Clinical and Molecular Medicine, Sapienza University of Rome, 00161 Rome, Italy; 6 SAFU Laboratory, Department of Research, Advanced Diagnostics and Technological Innovation, Translational Research Area, IRCCS Regina Elena National Cancer Institute, 00144 Rome, Italy; 7 Preclinical Models and New Therapeutic Agents Unit, IRCCS Regina Elena National Cancer Institute, 00144 Rome, Italy; 8 Unit of Melanoma, Cancer Immunotherapy and Development Therapeutics, Istituto Nazionale Tumori IRCCS ‘Fondazione G. Pascale’, 80131 Naples, Italy; 9 Faculty of Medicine and Psychology, Department Clinical and Molecular Medicine, Sant’Andrea Hospital, Sapienza University of Rome, 00118 Rome, Italy; 10 Scientific Directorate, IRCSS Regina Elena National Cancer Institute, 00144 Rome, Italy

**Keywords:** MicroRNA, Metastatic melanoma, Targeted therapy, Invasion, Migration

## Abstract

**Background:**

BRAF-mutant melanoma patients benefit from the combinatorial treatments with BRAF and MEK inhibitors. However, acquired drug resistance strongly limits the efficacy of these targeted therapies in time. Recently, many findings have underscored the involvement of microRNAs as main drivers of drug resistance. In this context, we previously identified a subset of oncomiRs strongly up-regulated in drug-resistant melanomas. In this work, we shed light on the molecular role of two as yet poorly characterized oncomiRs, miR-4443 and miR-4488.

**Methods:**

Invasion and migration have been determined by wound healing, transwell migration/invasion assays and Real Time Cell Analysis (RTCA) technology. miR-4488 and miR-4443 have been measured by qRT-PCR. Nestin levels have been tested by western blot, confocal immunofluorescence, immunohistochemical and flow cytometry analyses.

**Results:**

We demonstrate that the two oncomiRs are responsible for the enhanced migratory and invasive phenotypes, that are a hallmark of drug resistant melanoma cells. Moreover, miR-4443 and miR-4488 promote an aberrant cytoskeletal reorganization witnessed by the increased number of stress fibers and cellular protrusions-like cancer cell invadopodia. Mechanistically, we identified the intermediate filament nestin as a molecular target of both oncomiRs. Finally, we have shown that nestin levels are able to predict response to treatments in melanoma patients.

**Conclusions:**

Altogether these findings have profound translational implications in the attempt i) to develop miRNA-targeting therapies to mitigate the metastatic phenotypes of BRAF-mutant melanomas and ii) to identify novel biomarkers able to guide clinical decisions.

**Graphical Abstract:**

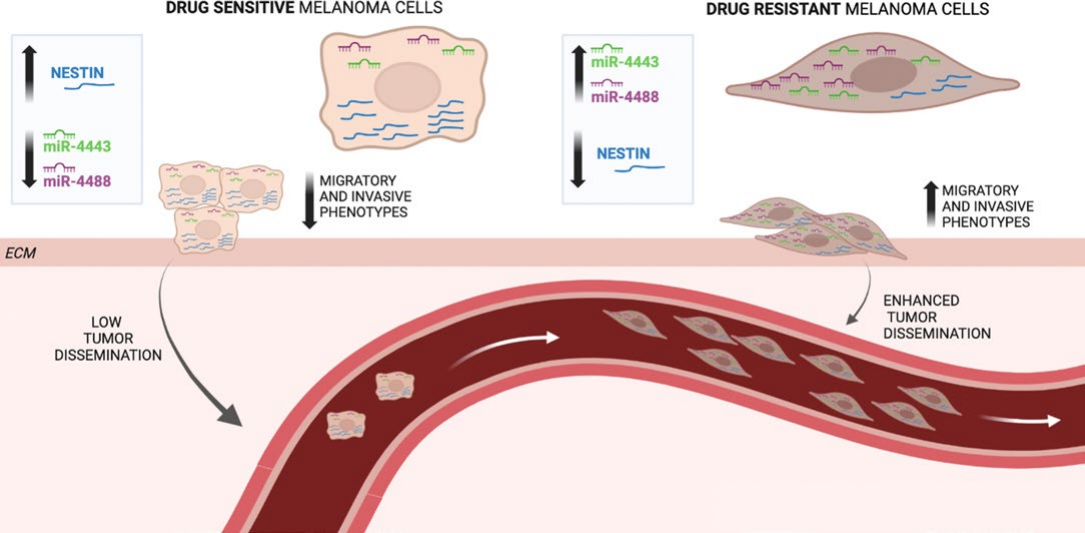

**Supplementary Information:**

The online version contains supplementary material available at 10.1186/s13046-023-02878-9.

## Background

Melanoma represents the most aggressive and lethal form of skin cancer [1]. During the last decades, the molecular characterization of this tumor has revealed the existence of four subtypes, namely BRAF mutated, NRAS mutated, NF1 mutated and triple *wild type* melanomas, respectively [2–4]. The detailed characterization of the functional alterations of BRAF mutated tumors and their dependencies from the constitutive activation of the BRAF/MEK/MAPK signaling pathway has paved the way to the development of targeted therapy [5]. BRAF mutated tumors still remain the only melanoma subtype that is treated with an approved targeted therapy. BRAF-mutations, which in most cases occur at position V600 with substitution of valine with glutamine (V600E), lead to the constitutive activation of the MAPK pathway which drives the alteration of different biological processes including cell proliferation, differentiation and stress responses [6]. Clinical efficacy of drugs able to inhibit the BRAF kinase is enhanced by the combined use of small molecules able to inhibit the downstream kinases MEK1 and MEK2 [7]. Nowadays three different combinations of BRAF and MEK inhibitors (MAPKi) have become the gold standard because they provide a significant improvement in objective responses and overall survival as compared to mono-therapy with BRAF inhibitors [8, 9]. Unfortunately, also combo targeted therapy is not immune from the development of drug resistance. Several studies have been directed to investigate the molecular mechanisms leading to acquired resistance to MAPKi in melanoma and allowed to identify both mutational and non-mutational events [10–16]. Among the latter types, growing relevance has been attributed to microRNAs (miRNAs) [17–22]. In this context, our group has contributed to uncover the involvement of a large number of miRNAs acting either as facilitators (i.e. oncomiRs) or antagonists (i.e. tumor suppressive miRNAs) of resistance, through whole miRnome profiling of melanoma cells undergoing the development of resistance to targeted therapies in vitro [23]. Among the most significantly deregulated miRNAs, we further explored the diagnostic and therapeutic properties of the two oncosuppressors miR-204-5p and miR-199b-5p, and of the two oncomiRs, miR-4443 and miR-4488. From the diagnostic standpoint, we studied the potential of these miRNAs to be used as non-invasive biomarkers to predict response to therapy in melanoma [24, 25]. Our results demonstrated that high circulating levels of miR-4488 are able to identify patients who develop a faster disease progression upon treatment with BRAF and MEK inhibiting drugs [26]. Of note, the best predictive results of disease outcome were obtained when miR-4488 is measured together with the oncosuppressive miR-579-3p and their ratio of expression is calculated [27]. Therapeutically, we demonstrated that enforcing expression of oncosuppressor miRNAs or inhibiting expression of oncomiRs was able to impair the emergence of resistance to MAPKi in vitro in long-term clonogenic assays [23]. Very recently, we further assessed the potential of miR-204-5p and miR-199b-5p to act as new therapeutics for melanoma when delivered systemically within lipid nanoparticles (LNP-miRs) [28]. Using this approach we successfully demonstrated that LNP-miRs are able to enhance the efficacy of targeted therapy in vivo*,* and to impair the development of de novo drug resistance [29]. Differently from the two oncosuppressor miRNAs mentioned above, the oncomiRs miR-4443 and miR-4488 have been so far poorly investigated. This has been the goal of the present work. By exploiting different biological assays, we demonstrate, here, that miR-4443 and miR-4488 contribute to the development of enhanced migratory and invasive phenotypes that are distinguishing characteristics of MAPKi resistant melanoma cells. Mechanistically, we demonstrate that this occurs via direct inhibition of the intermediate filament nestin. Reinstalling nestin expression in drug resistant BRAF mutated melanoma cells strongly impairs their migration and invasion ability.

## Methods

### Cell lines and transfection

BRAF-mutated melanoma cell lines A375 and LOX IMVI (V600E) were purchased from American Type Culture Collection (ATCC, Manassas, VA, USA). Human embryonic kidney 293 cells (HEK293) were purchased from System Bioscience (Palo Alto, CA, USA) and cultured according to the manufacturer’s instructions. Drug resistant A375 (RES) and LOX IMVI (RES) melanoma cells were selected by treating drug sensitive cells for about two months with increasing dabrafenib concentrations every two weeks from 50 nM to 2 μM. A375 double resistant cells (DRES) have been selected in the presence of both BRAF and MEK inhibitors as describe above. All human melanoma cell lines were cultured in RPMI (complete medium) supplemented with 10% (vol/vol) FBS, L-glu at 2% (vol/vol) and Pen-Strep at 1% (vol/vol). Sensitive A375 and LOX IMVI cell lines were plated at 70% confluence into 6 well plate in a RPMI with FBS at 10% and L-glu at 2%. Plated cells were transfected with 250 µl of Opti-MEM (Gibco, ThermoFisher, Waltham, MA, USA) mix composed by miRNAs-mimic or miRNAs-inhibitor mimic (Qiagen, Hilden, Germany) and a nestin plasmid (Origene, Rockville, MD, USA) at 0,1 µmol using respectively Lipofectamine [1000x] and Lipofectamine 3000 (ThermoFisher, Waltham, MA, USA). Transfected cells were subjected to the different experimental approaches described below.

### Wound healing assay

Wound healing assays were performed by using double well culture insert (Ibidi GmbH, Martinsried, Germany) as previously described [30]. Briefly, different melanoma cell lines transfected with SCR (scrambled), miR-4443 and miR-4488 or left untreated were plated into 6 well plates in a culture medium and cultured for 24 h a 37 °C, 5% CO2. Then, cells were trypsinized, counted and resuspended in 10% FBS RPMI. 3.5 × 10^4^ cells were placed into both wells of each insert with 90 μL of medium. After 24 h of culture, at confluence, the cell culture insert was removed. Each well was photographed at 10 × magnification immediately after insert removal (T-0) and after 24 h (T-24) with a Nikon DS-Fi1 camera (Nikon Corporation, Tokyo, Japan), coupled with a Zeiss Axiovert optical microscope (Zeiss, Oberkochen, Germany). The cell-free space (open residual area) was determined by using the ImageJ software (NIH, Rockville, USA).

### Migration assay

For cell migration assay, 5 × 10^4^ A375 and LOX IMVI melanoma cells transfected with SCR, miR-4443 or miR-4488 or left untreated were seeded in serum-free media into the upper chamber of Transwell (Corning, Costar, New York, USA) containing 8 µm pore polycarbonate membrane. The lower chamber was filled with 10% FBS RPMI. After 8 and 24 h of incubation at 37 °C respectively for each experiment, cells remaining on the top side of the membrane were removed using a cotton swab, and migrating cells were fixed, stained, photographed and counted [31]. Migrating cells were counted and the average number ± SD of cells are reported as fold change respect to control considered as 100. Three independent experiments were performed; each experiment was performed at least in quadruplicate.

### Invasion assay

For cell invasion assay, 5 × 10^4^ melanoma cells transfected with SCR, miR-4443 or miR-4488 or left untreated were seeded in serum-free media into the upper chamber of Transwell having a polycarbonate membrane coated with thin basement membrane (Corning® Matrigel® Basement Membrane Matrix, LDEV-free, 10 mL). The lower chamber was filled with 10% FBS RPMI. After 24 h of incubation at 37 °C, Matrigel and non-invading cells were mechanically removed with cotton swab. Polycarbonate filter containing invading cells was fixed with paraformaldehyde 4% in PBS (pH 7.4) at 4 °C and stained with Diff Quick solution. The filter was analyzed by optical microscope and four fields/filter were recovered at 10X magnification. Invading cells were counted and the average number ± SD of cells are reported as fold change respect to control considered as 100. Three independent experiments were performed; each experiment was performed at least in quadruplicate.

### RNA extraction and real-time PCR analysis

Total RNA was extracted using phenol–chloroform method (ThermoFisher Scientific) according to manufacturer’s instruction [32], and measured by fluorimetric assays using NanoDrop One C (ThermoFisher Scientific). RNA was retrotranscribed using the kit OneScript Plus cDNA Synthesis Kit (Abm, Richmond, BC, Canada) according to manufacturer’s instruction. The cDNAs obtained, were adequately diluted in DEPC-H_2_O. Following, 25 ng or 250 ng of cDNA were used to quantize the expression of miRNAs or genes respectively through real-time PCR using TaqMan Gene Expression Assays (ThermoFisher Scientific). Each targeted transcript was validated using the comparative Ct method for relative quantification (ΔΔCt). GADPH was used as a normalizer for genes, while U6 was used as a normalizer for miRNAs and the relative expression of each gene was determined using method 2 (-ΔΔCt).

### Real Time Cell Analysis (RTCA)

Cell migration and invasion were monitored in real time using the xCELLigence RTCA technology (Acea Bioscience, Santa Clara, CA, USA), as previously described [33]. To evaluate cell migration, sensitive and resistant A375 and LOX IMVI cells (2 × 10^4^ cells/well) were seeded in serum-free medium on filters in the upper chamber, which was located in a lower chamber that was filled with complete medium. Cell migration was monitored for the time range from 1 to 6 h, and each experiment was performed at least twice in quadruplicate. The slope represents the change rate of cell index values generated in a 0–1 h time frame. To evaluate cell invasion sensitive and resistant A375 and LOX IMVI cells (2 × 10^4^ cells/well) were seeded in serum-free medium on filters having a polycarbonate membrane coated with thin basement membrane (Corning® Matrigel® Basement Membrane Matrix, LDEV-free, 10 mL) in the upper chamber, which was located in a lower chamber that was filled with complete medium. Cell invasion was monitored for for the time range from 10 to 25 h, and each experiment was performed at least twice in quadruplicate. The slope represents the change rate of cell index values generated in a 0–1 h time frame.

### Western blot analysis

Cells were lysed with RIPA buffer (Sigma-Aldrich, San Louis, USA) and protein concentration was assessed using the Bradford assay (Biorad, Hercules, CA, USA). Equal amounts (30 μg/lane) of total protein were resolved under reducing conditions by SDS-PAGE (Bolt™ 4 to 12%, Bis–Tris, 1.0 mm, Mini Protein Gel, ThermoFisher Scientific) and transferred to reinforced nitrocellulose (BA-S 83, Schleider and Schuell, Keene, NH, USA). Membranes were blocked with 5% non-fat dry milk (Biorad) in TBS 0.1% (Sigma-Aldrich) Tween 20 (Sigma), and incubated with the different primary antibodies. Membranes were rehydrated and probed again with anti-GAPDH or anti-Tubulin, to estimate the protein equal loading. Densitometric analysis was performed using ImageJ software (NIH, Rockville, USA) and results were expressed as mean values from three independent experiments.

### Antibodies and reagents

Antibodies against nestin (for western blot, immunofluorescence and immunohistochemistry, IHC) and GAPDH were purchased from Cell Signaling Technology (Danvers, Massachusetts, USA) and from Santa-Cruz Biotechnology 8Dallas, Texas, USA), respectively. Antibody against nestin used for flow cytometry analysis was purchased from BD (Becton Dickinson S.p.a). TaqMan probes for U6, miR-4443, and miR-4488 were purchased from ThermoFisher Scientific. Dabrafenib and Trametinib were obtained from Novartis Farma S.p.A. (Rome, Italy).

### Immunofluorescence and confocal analysis

Immunofluorescence analysis was performed in A375 cells cultured into 8-well μ-slides (ibidi GmbH, AmKlopferspitz19, D-82152 Martinsried, Germany). After 48 h of culture cells were fixed with 4% paraformaldehyde for 10 min at 4 °C and washed twice for 10 min with PBS. Cells were permeabilized for 30 min using PBS, 3% Bovine serum albumin (BSA) (Santa Cruz Biotechnology), 0,1% Triton X-100 (Sigma-Aldrich), followed by mouse anti-nestin (Cell Signaling, Cat SC-73614 1:50 diluition) incubation at 4 °C o.n. After multiple washes with PBS, the cells were incubated with Alexa Fluor 594 anti-mouse secondary antibody (ThermoFisher Scientific) for 1 h at R.T. Actin filaments were stained with Alexa Fluor™ 488 Phalloidin (ThermoFisher Scientific). The slides were washed and incubated with the mounting media with DAPI (Vector Laboratories, Mowry Ave Newark, CA, USA). Finally, nestin expression levels were analyzed with Zeiss LSM 510 Meta confocal laser scanning microscope equipped with a 60X/1.23 NA oil immersion objective. Laser (488 and 514 nm), and HeNe laser (543 nm) were used to excite the fluorophores. The Zeiss Zen control software (Zeiss, Germany) was used for image analysis.

### Cell viability assay

5 × 10^4^ A375 cells were plated in a 96-wells plate. The number of viable drug sensitive vs resistant A375 melanoma cells was measured by quantification of the ATP present according to Cell Titer-Glo® Luminescent Cell Viability assay protocol (Promega, Madison, WI, USA) and luminescence was evaluated using GloMax® Explorer Multimode Microplate Reader (Promega).

### Flow cytometry analysis

The expression of nestin was also evaluated by flow cytometry analysis. After 48 h of plating 1 × 10^5^ of A375 or LOX IMVI cells were resuspended in 100 µL of PFA 1% and incubated for 20 min. Then, cells were washed in PBS and incubated in 0,1% Triton X-100 (Sigma-Aldrich) for 10 min. Cells were stained with and antibody PE mouse anti-Nestin for 45 min. Then, cells were resuspended in 300 µL 1X PBS (Sigma-Aldrich) and run to a flow cytometer. Non-stained cells were used as negative control. Approximately 10,000 events per sample were acquired with a CytoFLEX (Beckman Coulter, Milan, Italy) instrument equipped with three lasers (488 nm, 405 nm and 638 nm) and 9 detectors. Quality control of the cytometer was assessed daily using CytoFLEX Daily QC Fluorospheres (Beckman Coulter #B53230). Data were collected by CytExpert 2.2 version (Beckman Coulter) software. If needed, a compensation matrix was calculated using a VersaComp Antibody Capture Kit (Beckman Coulter #B22804) according to the manufacturer’s instructions. Data were analyzed using CytExpert version 2.2 software.

### Luciferase assay

Nestin as target of miR-4443 and miR-4488 was evaluated by luciferase assay. About 20 × 10^4^ HEK-293 cells were plated in a 6-wells plate. When cells reached 70% of confluence were co-transfected with plasmid containing the 3’ UTR of nestin (SC207843), miR-4443 or miR-4488 and Renilla luciferase plasmid (Promega). After 48 h of transfection, cells were processed using Dual-Luciferase® Reporter Assay System (Promega) and luminescence was evaluated using GloMax® Explorer Multimode Microplate Reader (Promega). The plasmid containing the 3′UTR of human nestin was purchased by Origene.

### IHC analyses

Melanoma biopsies fixed into 4% buffered formalin and paraffin embedded were subjected to IHC analyses to measure nestin levels using a specific antibody from Cell Signaling (Nestin clone 10C2, Mouse mAb #33,475) as previously reported [34]. Results were quantified counting positive cells in at least ten fields and expressed as percentage of positivity.

### Bioinformatics analyses and Kaplan–Meier curves

miR-4443 and miR-4488 putative targets with a binding score higher than 0.8 were predicted by miRWalk 3.0 (http://mirwalk.umm.uni-heidelberg.de/). miRNA binding sites were predicted within the complete sequence (5'-UTR, CDS and 3'-UTR) of the genes. The online software GEPIA 2.0 was interrogated to unveil nestin prognostic value based on Skin Cutaneous Melanoma (SKCM) data from The Cancer Genome Atlas (TCGA). Kaplan–Meier curves were plotted to assess the predictive values of nestin based on RNA-seq data coming from GSE65185 and IHC data coming from our internal cohort. The hazard ratio, Cox models and the log-rank *p* values were evaluated to plot KM curves.

### Ethical approval

The use of human samples was approved by Istituto Pascale’s Ethical Committee with the protocol DSC/2893 on April 11, 2015. All patients signed a general informed consent, which allowed the use of this material for research purposes in an anonymous manner.

### Statistical analysis

Experiments were replicated at least three times and the data were expressed as average ± SD or ± SE of the mean (SEM). Differences between groups were analyzed with a two-tailed paired or unpaired Student’s t-test or Mann–Whitney test and were considered statistically significant for *p*-value < 0.05.

## Results

### MAPKi-resistant melanoma cells show increased migratory and invasive phenotypes as compared to sensitive counterparts

**Fig. 1:**
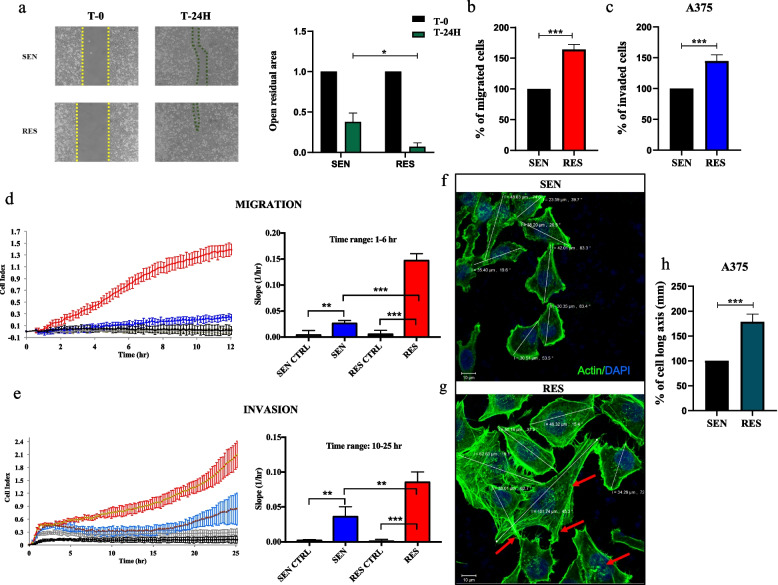
A375 resistant melanoma cells have increased migratory/invasive phenotypes and altered cytoskeletal remodeling as compared to sensitive counterparts. **a** A375 sensitive cells (SEN) and their resistant counterparts (RES) were cultured in the presence of double well culture inserts to perform wound healing assays. After 24 h, inserts were removed and each well was photographed at 10 × magnification (scale bar 500 μm) immediately after insert removal (T-0) and after 24 h (T-24H) (left panel). The open residual area (right panel) was measured by using the ImageJ software in RES cells as compared to SEN ones. The values were calculated as “fold change” (± SD). **b** To perform migration assays, A375 SEN and RES cells were seeded in serum-free media into the upper chamber of a transwell, whereas the lower chamber was filled with 10% FBS RPMI. After 8 h, cells remaining on the top side of the membrane were counted and the average number ± SD of cells are reported as fold change respect to control considered as 100. **c** To perform invasion assays, A375 SEN and RES cells were seeded into the upper chamber of a transwell coated with Matrigel, whereas the lower chamber was filled with 10% FBS RPMI. After 24 h, invading cells were counted and the average number ± SD of cells are reported as fold change respect to control considered as 100. Three independent experiments were performed (**a**, **b**, **c**); each experiment was performed at least in quadruplicate. **d**, **e** xCELLigence RTCA technology was used to measure cell migration and invasion of A375 SEN and RES cells. To evaluate migration (**d**), cells were seeded in serum-free medium on filters in the upper chamber, whereas the lower chamber was filled with 10% FBS RPMI. Cell migration was monitored for 6 h. To evaluate invasion (**e**), cells were seeded in serum-free medium on filters coated with Matrigel in the upper chamber, whereas the lower chamber was filled with 10% FBS RPMI. Cell invasion was monitored for 25 h. Each experiment was performed at least twice in quadruplicate (**d**, **e**). The slope represents the change rate of cell index values generated in a 0–1 h time frame. **f**, **g** Representative images by confocal microscopy of F-Actin and DAPI immunostaining of A375 SEN and RES cells that were cultured into 8-well μ-slides for 48 h. Red arrows indicate filopodia and stress fibers. Magnification 63x. **h** Quantification analyses of the long cell axis measured from the aforementioned confocal images were performed by Zeiss Zen control software. All the experiments have been performed at least in triplicate ± standard deviation (SD) and *p*-value < 0.05 was considered as significant (Student’s t-test)

It has been previously reported that melanoma cells rendered resistant to MAPKi are characterized by increased cell motility as compared to sensitive counterparts [35–37]. However, the molecular mechanisms behind this have not yet been clearly elucidated. Our previous results underscored that migration/invasion phenotypes are among the top molecular pathways governed by miRNAs found deregulated in drug-resistant melanoma cells [23]. Therefore, we decided to investigate this potential crosstalk. We first tested whether drug resistant melanoma cells previously selected in our lab [27–29] were characterized by increased migratory and invasive capabilities as compared to sensitive counterparts. This was accomplished using different biological assays. First of all, we performed wound healing assays using A375 melanoma cells. Results demonstrated that drug-resistant A375 cells are characterized by an enhanced migration capability as compared to their sensitive counterparts (Fig. 1a). To exclude that the increased migratory capability of resistant cells was due to increased proliferation we tested cell viability at different time points. Data shown in Suppl. Figure 1a demonstrate that the proliferative rate is not different between sensitive vs drug resistant cells. We then moved to confirm our findings using transwell migration assays. Results confirmed that A375 resistant cells are characterized by an increased migration rate as compared to sensitive counterparts (Fig. 1b). Besides the evaluation of cell migration, we have also tested the invasive capability of drug resistant melanoma cells, demonstrating that drug-resistant A375 cells are also more invasive as compared to their sensitive counterparts (Fig. 1c). These results were also confirmed in two additional resistant melanoma cell lines, namely LOX IMVI RES and A375 DRES to both BRAFi and MEKi (Suppl. Figure 1b-e). Moving forward, we decided to strengthen our data by exploiting the advanced technology of xCELLigence RTCA, which records as cell index the changes of impedance due to the adhesion of migrating/invading cells to microelectrodes. The representative graphs recorded for 6 h show that A375 drug resistant cells are characterized by a strongly increased migratory capability as compared to drug sensitive cells (Fig. 1d). Furthermore, the same technology was also used to measure the invasive phenotype of melanoma cells testing for 25 h, their ability to soak Matrigel membranes. Also in this case, drug resistant cells demonstrated to be more invasive as compared to sensitive counterparts (Fig. 1e). Of note, the same results obtained by xCELLigence RTCA technology were obtained also in LOX IMVI RES and A375 DRES cells (Suppl. Figure 2a-d). We reasoned that the increased migratory and invasive phenotypes of drug resistant melanoma cells may be potentially related to an altered cytoskeletal remodeling of these cells as compared to sensitive counterparts [38]. To this aim, we performed confocal immunofluorescence analyses based on the staining of one of the major cytoskeletal proteins, namely actin. Interestingly, our results demonstrated that A375 drug resistant cells are characterized by a more elongated morphology as compared to sensitive cells, as underscored by the measures of the long cell axis (Fig. 1f-h). Moreover, resistant cells also showed an increased number of stress fibers and cellular protrusions-like lamellipodia, filopodia and invadopodia as compared to sensitive counterparts (see red arrows in Fig. 1g). These results were confirmed also in A375 DRES cells (Suppl. Figure 3). This actin-cytoskeleton re-organization is consistent with the enhanced migratory and invasive phenotypes of resistant cells highlighted by all the biological assays described above.

### miR-4443 and miR-4488 overexpression triggers the migration and invasive potential of melanoma cells

**Fig. 2:**
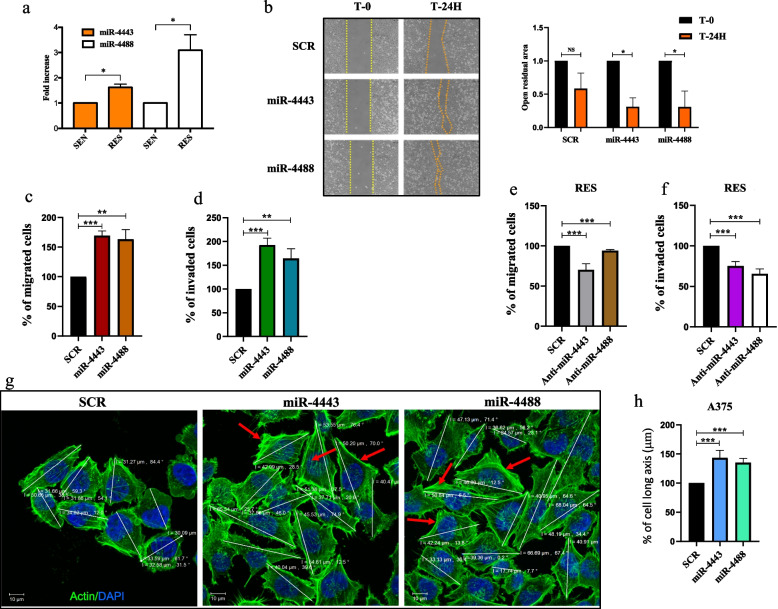
miR-4443 and miR-4488 transient overexpression affects migration/invasive potential and cytoskeletal remodeling of melanoma cells. **a** Total RNAs were extracted from A375 sensitive cells (SEN) and their resistant counterparts (RES) to perform qRT-PCR analyses for miR-4443 and miR-4488 expression levels. Each miRNA transcript was quantified using the comparative Ct method for relative quantification (ΔΔCt) using U6 as normalizer. The values were calculated as “fold change” (± SD) compared to SEN considered as 1. **b** A375 SEN cells were transiently transfected with miR-4443, miR-4488 or the relative scrambled (SCR) for 48 h. Transfected cells have been then harvested and cultured in the presence of double well culture inserts to perform wound healing assays. After 24 h, inserts were removed and each well was photographed at 10 × magnification (scale bar 500 μm) immediately after insert removal (T-0) and after 24 h (T-24H) (left panel). The open residual area (right panel) was measured by using the ImageJ software in oncomiR-transfected cells as compared to SCR-transfected ones. The values were calculated as “fold change” (± SD). **c** To perform migration assays, A375 SEN cells, transfected as described above, were seeded in serum-free media into the upper chamber of a transwell, whereas the lower chamber was filled with 10% FBS RPMI. After 8 h, cells remaining on the top side of the membrane were counted and the average number ± SD of cells are reported as fold change respect to control (SCR) considered as 100. **d** To perform invasion assays, A375 SEN cells, transfected as described above, were seeded into the upper chamber of a transwell coated with Matrigel, whereas the lower chamber was filled with 10% FBS RPMI. After 24 h, invading cells were counted and the average number ± SD of cells are reported as fold change respect to control (SCR) considered as 100. **e**, **f** Migration and invasion assays were performed as described above using A375 RES cells transiently transfected with the anti-miR-4443, the anti-miR-4448 or the SCR for 48 h before performing the appropriate assays. For all migration and invasion assays, three independent experiments were performed; each experiment was performed at least in quadruplicate. **g** Representative images by confocal microscopy of F-Actin and DAPI immunostaining of A375 SEN cells transiently transfected with miR-4443, miR-4488 or the SCR for 48 h. Red arrows indicate filopodia and stress fibers. Magnification 63x. **h** Quantification analyses of the long cell axis measured from the aforementioned confocal images were performed by Zeiss Zen control software. All the experiments have been performed at least in triplicate ± standard deviation (SD) and *p*-value < 0.05 was considered as significant (Student’s t-test)

In the previous paragraph, we have showed that MAPKi-resistant melanoma cells have superior migratory and invasive capabilities as compared to their drug sensitive counterparts. Next, we went further to demonstrate a link between this phenotype and the deregulation of the oncomiRs previously discovered by our group, namely miR-4443 and miR-4488. First of all, we confirmed by qRT-PCR the upregulation of these miRNAs in A375 and LOX IMVI drug resistant cells (Fig. 2a and Suppl. Figure 4a) in line with our previous findings [23]. We then decided to test whether the enforced expression of miR-4443 or miR-4488 in drug sensitive melanoma cells could be able to increase the migratory and invasive phenotype of drug-resistant melanoma cells. To this aim, A375 and LOX IMVI sensitive cells were transiently transfected for 48 h with oncomiR mimics or the relative scrambled (SCR) sequences as controls. Results obtained by qRT-PCR confirmed the strong upregulation of both miR-4443 and miR-4488 in the two cell lines tested (Suppl. Figure 4b,c). Then, we started to evaluate the effects of these upregulations on migratory and invasive capabilities of transfected cells. This was accomplished using different experimental approaches: wound healing and transwell migration assays performed as described above. Starting from the first approach, our results clearly demonstrated that A375 drug-sensitive cells transiently transfected with miR-4443 or miR-4488 mimics have superior migratory capabilities as compared to SCR-transfected cells (Fig. 2b). These data were then confirmed using transwell migration assays. Accordingly, oncomiR-transfected A375 and LOX IMVI melanoma cells were able to better migrate through membrane pores as compared to SCR-transfected cells (Fig. 2c and Suppl Fig. 4d). Moreover, we have also evaluated the impact of miR-4443 and miR-4488 overexpression on the induction of the invasive phenotypes of drug sensitive melanoma cells. To this aim, oncomiR-transfected cells were seeded into the upper chamber of transwell coated with a thin membrane of matrigel. Results clearly show A375 and LOX IMVI cells transiently transfected with miR-4443 and miR-4488 mimics were more invasive as compared to SCR-transfected cells (Fig. 2d and Suppl Fig. 4e). To strengthen these findings, we evaluated also the effects of miR-4443 and miR-4488 silencing on the migratory and invasive capabilities of A375 drug-resistant melanoma cells. Results obtained upon 48 h of anti-miR-4443 or anti-miR-4488 transient transfection clearly demonstrated that drug-resistant melanoma cells in which the two oncomiRs have been silenced are characterized by lower migration and invasion capabilities as compared to SCR transfected cells (Fig. 2e,f). Finally, we decided to evaluate by confocal immunofluorescence analyses the morphological features of miR-4443 and miR-4488-transfected A375 sensitive cells. Results showed that these cells are characterized not only by a more elongated shape, but also by an increased number of stress fibers and structures like invadopodia as compared to SCR-transfected cells (Fig. 2g,h). Of note, this altered cytoskeletal remodeling resembles what observed in A375 drug resistant melanoma cells (Fig. 1f-h). Altogether, these data demonstrate that miR-4443 and miR-4488 are able to trigger the migratory and invasive capabilities of drug sensitive melanoma cells in vitro by inducing the reshaping of their cellular architecture.

### The intermediate filament nestin is a molecular target of miR-4443/miR-4488 in melanoma

**Fig. 3:**
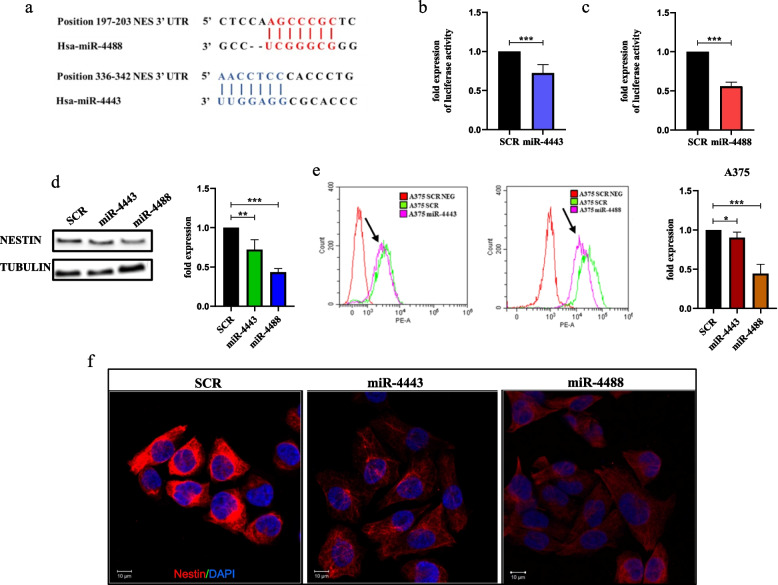
The intermediate filament Nestin is a molecular target of miR-4443 and miR-4488 in melanoma. **a** Schematic representation of the entire 3’UTR of mRNA relative to nestin containing the two seed regions for miR-4488 and for miR-4443 located at positions 197–203 and 336–342, respectively (source miRWalk3.0). **b**, **c** To perform luciferase assays, HEK-293 cells were plated in a 6-wells plate and then co-transfected with a plasmid containing the 3’ UTR of nestin and Renilla luciferase plasmid together with miR-4443 (**b**), miR-4488 (**c**) or the relative scrambled (SCR). After 48 h of transfection, cells were harvested and luminescence was evaluated using Dual-Luciferase® Reporter Assay System. The values were calculated as fold change (± SD) compared to SCR considered as 1. **d** A375 SEN cells were transiently transfected with miR-4443, miR-4488 or with the SCR for 72 h. Cells have been then harvested and lysed to extract total proteins that have been subjected to Western blot analyses with the indicated antibodies (left panel). Tubulin levels have been estimated for the protein equal loading and densitometric analyses were performed using ImageJ software (right panel). Results were expressed as mean values from three independent experiments. The values were calculated as fold change (± SD) compared to SCR considered as 1. **e** A375 SEN cells transfected as described above have been stained with an antibody PE mouse anti-nestin for 45 min to perform flow cytometry analyses. Non-stained cells were used as negative control. Black arrows indicate the peaks relative to cells transfected with miR-4443 or miR-4488 mimics (left graphs). Quantitative analyses were calculated as “fold change” (± SD) compared to SCR considered as 1 (right graph). Data were analyzed using CytExpert version 2.2 software. **f** Representative images by confocal microscopy of nestin and DAPI immunostaining of A375 SEN cells transiently transfected as described above. Magnification 63x. All the experiments have been performed at least in triplicate ± standard deviation (SD) and *p*-value < 0.05 was considered as significant (Student’s t-test)

In the previous sections we have shown that 1) drug resistant melanoma cells are endowed with enhanced migratory and invasive capabilities as compared to their drug sensitive counterparts; 2) miR-4443 and miR-4488 overexpression in sensitive cells is able to phenocopy the metastatic potential of drug resistant cells; 3) an aberrant cytoskeletal remodeling is shared by drug-resistant cells and oncomiR-transfected sensitive melanoma cells. In order to unravel the mechanism of action of the two oncomiRs we moved to identify the molecular targets of miR-4443 and miR-4488 potentially involved in this process. To this purpose, we performed bioinformatics analyses, using the online tool miRWalk3.0 [39], to identify the potential target genes of both the two oncomiRs. Interestingly, we found that the intermediate filament nestin has two potential seed regions for these miRNAs within its 3’UTR located at positions i) 197–203 for miR-4488 and ii) 336–342 for miR-4443 (Fig. 3a). These predictions attracted our attention because nestin has been reported to be involved in 1) the regulation of migratory and invasive phenotypes of cancer cells and 2) the cytoskeletal re-organization in different cell subsets [40, 41]. To confirm these predictions, the nestin 3’UTR containing miR-4488 and miR-4443 binding sites was cloned downstream of the luciferase Open Reading Frame (ORF) in a reporter plasmid. This construct was transiently transfected in HEK-293 cells together with miR-4443, miR-4488 mimics or SCR sequences as controls. In line with in silico predictions, we observed a significant reduction of luciferase activity in cells transfected with oncomiRs as compared to the negative controls (Fig. 3b,c). Then we moved to validate luciferase assay data in the in vitro melanoma models. To this aim, nestin expression was analyzed in A375 cells transiently transfected with oncomiR mimics or SCR sequences. Results of western blotting and flow cytometry analyses clearly showed a significant decrease of nestin expression levels in cells transfected with miR-4443 or miR-4488 as compared to their SCR-transfected counterparts. Representative blots, flow cytometry panels and the relative quantitative analyses are reported in Fig. 3d,e. These results were also confirmed by flow cytometry in LOX IMVI cells (Suppl. Figure 5a). It has to be mentioned that in these cells nestin relative protein was not detectable by western blotting analyses (Suppl. Figure 5b) probably because its basal levels are lower as compared to A375 cells. Therefore, we decided to evaluate nestin expression in LOX IMVI cells with a more sensitive technique, namely flow cytometry assay. Finally, we confirmed the downregulation of nestin in miR-4443- and miR-4488-transfected A375 sensitive cells by confocal immunofluorescence analyses (Fig. 3f). Altogether these data allowed to define nestin as novel molecular target of miR-4443/miR-4488 in melanoma. The deregulation of this axis may explain the increased migratory and invasive phenotypes of our drug resistant melanoma models as compared to sensitive counterparts.

### Nestin levels are lost in drug resistant melanoma cells and predict response to MAPKi in patients

**Fig. 4:**
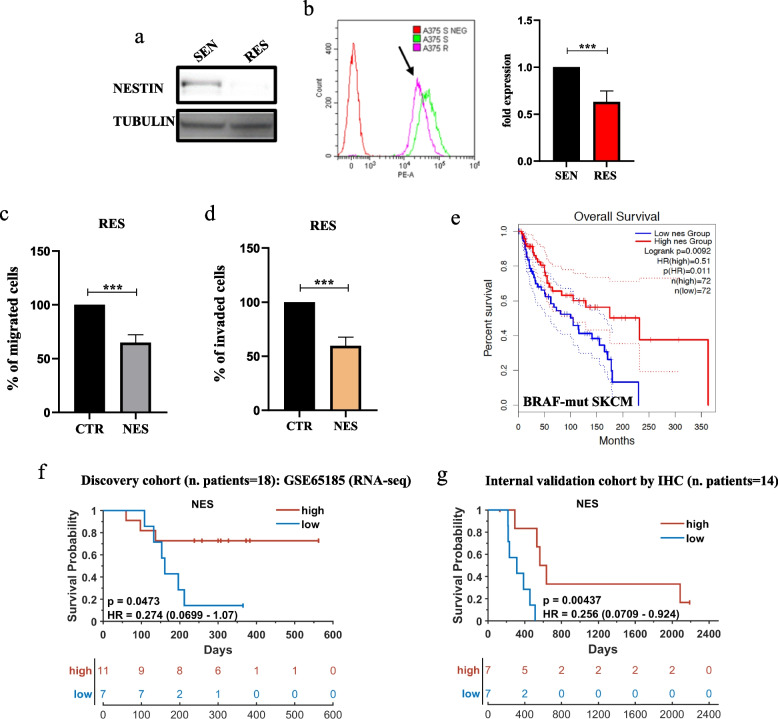
Nestin levels are reduced in drug resistant melanoma cells and are associated with targeted therapy response in patients. **a** A375 SEN and RES cells were harvested and lysed to extract total proteins to perform Western blot analyses with the indicated antibodies. Tubulin levels have been estimated for the protein equal loading. **b** A375 SEN and RES cells have been stained with an antibody PE mouse anti-nestin for 45 min to perform flow cytometry analyses. Non-stained cells were used as negative control. Black arrow indicates the peaks relative to RES cells (left graph). Quantitative analyses were calculated as “fold change” ± standard deviation (SD) compared to SEN cells considered as 1. Data were analyzed using CytExpert version 2.2 software. **c**, **d** Migration and invasion assays were performed as described above using A375 RES cells transiently transfected with a plasmid encoding for nestin (NES) or the relative empty control (CTR) for 48 h. Results have been quantified by counting cells remaining on the top side of the membrane and the average number ± SD of cells are reported as fold change respect to control (SCR) considered as 100. **e **The online software GEPIA 2.0 (http://gepia2.cancer-pku.cn/#index) was interrogated to unveil nestin prognostic value based on Skin Cutaneous Melanoma (SKCM) data from The Cancer Genome Atlas (TCGA). Patients have been stratified according to the mutational status and the Kaplan Meier (KM) curves represent the data relative to the BRAF-mutant subtype (*n* = 149). **f** The mRNA levels relative to nestin (NES) have been extracted from bulk RNA-seq data deriving from 18 melanoma patients before starting MAPKi therapy (GSE65185) deposited in Gene Expression Omnibus (GEO) database. Data have been used to plot KM curves to assess the predictive value of NES for therapy response (Discovery cohort). **g** NES levels were evaluated by immunohistochemistry (IHC) in 14 melanoma patients’ biopsies collected before starting MAPKi therapy. Data have been used to plot KM curves to assess the predictive value of NES for therapy response (internal validation cohort). High and low levels were assessed by considering positive and negative z-scores, respectively. The hazard ratio, Cox models and the log-rank *p* values were evaluated to plot KM curves

**Fig. 5:**
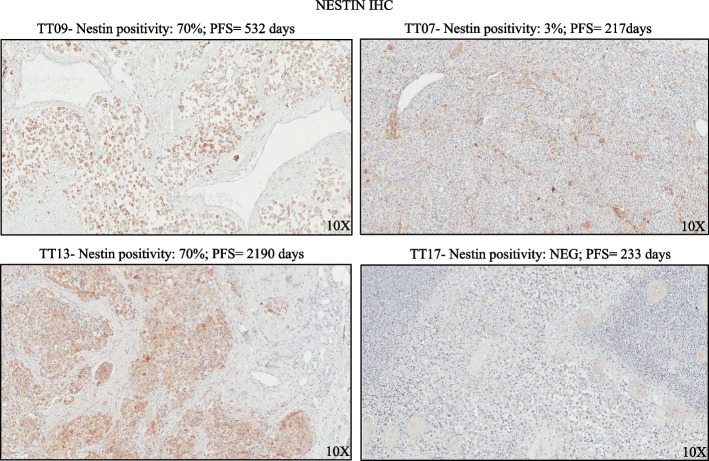
Nestin levels in representative melanoma patients. Melanoma biopsies collected before starting MAPKi therapy were subjected to immunohistochemistry (IHC) analyses to measure nestin levels. Results were quantified counting positive cells in at least ten fields and expressed as percentage of positivity. Representative images of four cases are reported (TT07, TT09, TT13, and TT17). 10X magnification (scale bar 500 μm). Progression Free Survival (PFS) is expressed in days. All the clinical data are available in Suppl. Table 1

Lastly, we decided to measure the levels of nestin in in vitro melanoma drug resistant cell lines. Results shown in Fig. 4a,b demonstrated that nestin is strongly reduced in A375 RES cells. Of note, the same findings have been obtained also in LOX IMVI RES and in A375 DRES cells (Suppl. Figure 5c,d and Suppl. Figure 6a,b). Moving forward, we sought to demonstrate a direct connection between the downregulation of nestin and the enhanced migratory and invasive phenotypes of resistant cells. To this aim, we transiently transfected A375 resistant cells with an expression vector coding for nestin and carried out Boyden chamber and Transwell invasion assays. Results demonstrated that reinstalling nestin levels in resistant cells was able to decrease their migratory and invasive properties as compared to cells transfected with control plasmid (Fig. 4c,d). All the data shown above suggest a potential anti-invasive role of nestin in melanoma. To answer this point, we have evaluated the nestin prognostic value in TCGA public available melanoma dataset using the online software GEPIA [42], which uses the data deposited on The Cancer Genome Atlas (TCGA). Interestingly, our results unveiled that high levels of nestin are associated with a better survival in patients harboring BRAF mutations (Fig. 4e). Of note, this correlation was not evident in the other mutational subtypes, i.e. NF1, RAS and Triple WT (Suppl. Figure 7a), thus suggesting a specificity of nestin prognostic value for BRAF-mutant melanomas. Moreover, we asked whether nestin levels may be also predictive of response to targeted therapies in melanoma patients. To this aim we analyzed public RNA-seq data available in the Gene Expression Omnibus (GEO) database under the accession code GSE65185 [43]. This dataset contains gene expression data coming from melanoma biopsies sequenced before starting targeted therapies. Kaplan–Meier curves clearly demonstrate that patients with higher levels of nestin were characterized by a longer progression free survival (PFS) as compared to patients with lower levels of nestin (Fig. 4f). To strengthen these observations, we have analyzed nestin protein levels by IHC in an internal cohort composed of 14 BRAF-mutant melanoma patients whose biopsies have been collected before starting targeted therapies. Results confirm that nestin high levels are predictive of a better PFS for melanoma patients (Fig. 4g). The complete results of IHC together with the clinical characteristics of the patients included in our analysis are reported in Suppl. Table 1, whereas representative images of nestin expression are shown in Fig. 5. Of note, in the same samples we have also analyzed the levels of miR-4443/miR-4488 by qRT-PCR to assess whether the combination of nestin with the two oncomiRs was able to achieve a stronger predictive value for PFS. Results, shown as Kaplan–Meier curves, demonstrated only a slight improvement of p- and Hazard ratio (HR) values in nestin + miRNAs as compared to nestin alone (*p* = 0.00315; HR = 4.1 vs *p* = 0.00437; HR = 0.256) (Suppl. Figure 7b). Altogether these findings underscore not only a potential oncosuppressive role for nestin in BRAF-mutant melanomas but also unveil its potential as a predictive marker of clinical response to targeted therapy.

## Discussion

In this paper we shed light on the molecular mechanisms governed by a newly identified couple of oncomiRs, namely miR-4488 and miR-4443, in BRAF-mutant melanomas. These miRNAs have been previously identified by our group to act as facilitators of the acquisition of resistance to targeted therapy in melanoma [23]. The focus of the present study was to investigate in details the biological effects triggered by these two miRNAs and their mechanism of action. By exploiting a combination of different biological assays, we demonstrated that miR-4443 and miR-4488 are able to govern the migratory and invasive phenotypes of drug resistant melanoma cells. Despite these features have been already described to be a hallmark of melanoma cells upon acquisition of resistance to targeted therapies in melanoma [35–37, 44, 45], the main novelty of our work is to have linked these phenotypes to the deregulation of the two specific oncomiRs. Moreover, these findings are in line with our previous analyses which underscored that the rewiring of migration- and invasion-related pathways are among the main molecular avenues governed by the miRNAs found altered between sensitive vs resistant melanoma cells [22]. So far whereas miR-4443 aberrant expression has been reported in different human diseases [46], including cancer, miR-4488 has been the objects of a limited number of studies. Of note, neither of these two miRNAs has never been investigated in melanoma. For example, it has been demonstrated that miR-4443 promotes the metastatic properties of breast cancer and esophageal squamous cell carcinoma by suppressing the tissue inhibitors of metalloproteinase 2 (TIMP2) [47–50]. Moreover, this miRNA has been also found to induce chemo- and radio-therapy resistance in non-small cell lung cancer and esophageal squamous cell carcinoma [51–53]. However, these works are substantially different from our data because they i) have been focused on different tumor types and ii) unveiled molecular mechanisms totally different from the results presented here. In opposition to the aforementioned studies, other works have demonstrated that miR-4443 may act as an oncosuppressive miRNA in osteosarcoma, colon cancer and hepatocellular carcinoma [54–57]. Finally, it has been also reported that this miRNA is a non-canonical small RNA because it is not produced via Drosha–Exportin 5–Dicer pathway but, differently, by a yet unknown biogenesis pathway [58]. Regarding miR-4488, although this miRNA has been reported to be aberrantly expressed in different tumor types, like leukemia [59] and colorectal cancer [60], its mechanism of action, as well as its target genes, have never been identified prior to our present study. Interestingly, different studies converge in describing miR-4488 as an exosomal specific miRNA. Indeed, miR-4488 has been found to be enriched in melanoma- and breast cancer-derived exosomes [61, 62] and, thereby, potentially acting as an extracellular messenger of oncogenic signals from tumors cells to the other cellular elements residing in the tumor microenvironment [63]. In line with this, we have recently demonstrated that the higher circulating levels of miR-4488 are able to distinguish melanoma patients who develop faster progression of disease upon first line therapies with MAPKi [26]. Further studies are needed to unveil whether drug resistant melanoma cells may potentially release higher levels of exosomes loaded with miR-4488 as compared to sensitive counterparts. Besides the biological effects of miR-4443 and miR-4888, the other aspect of interest of our study is to have demonstrated that the enforced expression of these miRNAs in sensitive melanoma cells is able to phenocopy the altered cytoskeletal remodeling that is a peculiar characteristic of drug resistant melanoma cells. This is witnessed by an altered actin remodeling that induces a more elongated cell shape, peculiar of a migratory phenotype, in parallel with the aberrant formation of stress fibers and protrusions like tumor cell invadopodia. Coherently, these structures are the major conductors that orchestrate cancer cell metastatization [64]. Furthermore, drug resistant melanoma cells, as well as sensitive ones, after transient overexpression of miR-4443/miR-4488 share an increased cells size which probably relies to an increased cytoskeletal tension and stiffness. Of note, this peculiar cellular architecture has been previously described in melanoma cells rendered resistant to MAPKi [65] despite never correlated to be affected by miRNAs. The other aspect of interest of our study is to have identified the intermediate filament nestin as a molecular target of both miR-4443/miR-4488. These findings are of interest for several reasons. First of all, nestin levels are lost in melanoma cells upon acquisition of targeted therapy resistance in contrast to miR-4443/miR-4488 that are up-regulated. Of note, the reduction of nestin in drug resistant cells have been already reported by other investigators [66] despite, again, never explained at molecular level like in our case, i.e. to be regulated by specific oncomiRs. Moreover, in line with our findings, nestin inhibition has been reported to be sufficient to increase melanoma cell invasiveness in vitro [66]. Interestingly, single cell analyses by mass cytometry have demonstrated that BRAFi and MEKi treatments are able to selectively kill nestin-expressing melanoma cells [67]. These findings may explain why drug resistant melanoma cells that have been challenged by targeted therapy are characterized by the loss of nestin. It has to be mentioned that the oncosuppressive role of nestin in melanoma is challenged by different and contrasting studies that have reported an oncogenic role for this protein [68, 69], not only in melanoma, but also in colorectal and hepatocellular carcinomas [70, 71]. However, we believe that the real breakthrough of our work is to have attributed a novel role for nestin, namely to be a prognostic biomarker in the specific BRAF-mutant subtype, as demonstrated by nestin association with better PFS only in this mutational subtype. In addition, the possibility to stratify patients with BRAF-mut melanomas for a longer benefit of the therapy with BRAFi and MEKi is challenging and warrants of further validation in an enlarged cohort of patients. Most importantly, it may be also possible to combine data of nestin expression in solid biopsies with the circulating levels of miR-4443 and miR-4488 in the same patients to elaborate even more accurate and specific models of prediction of response to treatments. Indeed, our recent studies, especially in the case of miR-4488, have proved the capability of circulating miRNAs to distinguish patients who develop a faster disease progression upon treatment with BRAF and MEK inhibiting drugs.

Besides the potential diagnostic implications of our study, the latter aspect of interest of our study relies on the possibility to therapeutically translate these findings. In this context, a possibility will be provided by the use of locked nucleic acid (LNA)-modified antimiRs which have been developed to inhibit the expression of oncogenic miRNAs [72]. This strategy has been widely reported to be effective in different in vitro and in vivo preclinical models [73]. For example, the intravenous injection in murine cancer models of LNAs targeting miR-21 have demonstrated anti-tumor effects in breast cancer [74], whereas miR-17 and miR-21 targeting has demonstrated effectiveness in medulloblastoma models [75]. Therefore, we plan to design specific LNAs to inhibit the expression levels of miR-4443 and miR-4488 to reduce to metastatic properties of drug resistant melanoma cells and, potentially, also to restore drug sensitivity. In this context, we have recently reported that miRNA-based therapy may be a successful strategy to improve MAPKi efficacy in BRAF-mutant melanoma both in vitro and in vivo. This has been accomplished using LNPs to reinstall the balance of oncosuppressive miRNAs, like miR-204-5p and miR-199b-5p, in different drug sensitive and resistant melanoma models [29].

## Conclusions

Overall, we believe that our findings are of interest 1) at translational level because our results suggest that the targeting of miR-4443/miR-4488 may be a therapeutic strategy to tackle the metastatic potential of MAPKi-resistant melanoma cells and 2) at a mechanistic level because we have identified nestin as a novel target of both oncomiRs. Finally, the evidence that nestin expression level is able to predict therapy response in melanoma has profound clinical implications in the attempt to identify novel biomarkers able to guide clinical decisions in the management of this aggressive disease.

### Supplementary Information

**Additional file 1: Suppl. Fig. 1.** a. A375 sensitive cells (SEN) and their resistant counterparts (RES) were plated in a 96-wells plate. The number of viable cells was measured by quantification of the cellular ATP present at different time points (from 24 to 96 hours). Results were calculated as fold change (± SD) relative to the time point of 24 hours. b, c. To perform migration assays, LOX IMVI SEN and RES cells or A375 SEN and DRES cells (double resistant to BRAFi and MEKi) were seeded in serum-free media into the upper chamber of a transwell, whereas the lower chamber was filled with 10% FBS RPMI. After 8 hours, cells remaining on the top side of the membrane were counted and the average number ± SD of cells are reported as fold change respect to control considered as 100. d, e. To perform invasion assays, LOX IMVI SEN and RES cells or A375 SEN and DRES cells were seeded into the upper chamber of a transwell coated with Matrigel, whereas the lower chamber was filled with 10% FBS RPMI. After 24 hours, invading cells were counted and the average number ± SD of cells are reported as fold change respect to control considered as 100. Three independent experiments were performed; each experiment was performed at least in quadruplicate. All the experiments have been performed at least in triplicate ± standard deviation (SD) and p-value < 0.05 was considered as significant (Student’s t-test). **Suppl. Fig. 2.** a-d. xCELLigence RTCA technology was used to measure cell migration and invasion of LOX IMVI SEN and RES cells or A375 SEN and DRES cells (double resistant to BRAFi and MEKi). To evaluate migration (a, c), cells were seeded in serum-free medium on filters in the upper chamber, whereas the lower chamber was filled with 10% FBS RPMI. Cell migration was monitored for 8 hours. To evaluate invasion (b, d), cells were seeded in serum-free medium on filters coated with Matrigel in the upper chamber, whereas the lower chamber was filled with 10% FBS RPMI. Cell invasion was monitored for 18 hours. Each experiment was performed at least twice in quadruplicate. The slope represents the change rate of cell index values generated in a 0–1 hour time frame. All the experiments have been performed at least in triplicate. **Suppl. Fig. 3.** Representative images by confocal microscopy of F-Actin and DAPI immunostaining of A375 SEN and DRES cells (double resistant to BRAFi and MEKi) that were cultured into 8-well μ-slides for 48 hours (left panels). Magnification 63x. Quantification analyses of the long cell axis measured from the aforementioned confocal images were performed by Zeiss Zen control software (right graph). All the experiments have been performed at least in triplicate ± standard deviation (SD) and p-value < 0.05 was considered as significant (Student’s t-test). **Suppl. Fig. 4.** a. Total RNAs were extracted from LOX IMVI sensitive cells (SEN) and their resistant counterparts (RES) to perform qRT-PCR analyses for miR-4443 and miR-4488 expression levels. Each miRNA transcript was quantified using the comparative Ct method for relative quantification (ΔΔCt) using U6 as normalizer. The values were calculated as “fold change” (± SD) compared to SEN considered as 1. b, c. A375 SEN and LOX IMVI SEN cells were transiently transfected with miR-4443, miR-4488 or the relative scrambled (SCR) for 48 hours. Cells have been then harvested and the expression levels of miR-4443 and miR-4488 was determined as described above. d. To perform migration assays, LOX IMVI SEN cells, transfected as described above, were seeded in serum-free media into the upper chamber of a transwell, whereas the lower chamber was filled with 10% FBS RPMI. After 8 hours, cells remaining on the top side of the membrane were counted and the average number ± SD of cells are reported as fold change respect to control (SCR) considered as 100. e. To perform invasion assays, LOX IMVI SEN cells, transfected as described above, were seeded into the upper chamber of a transwell coated with Matrigel, whereas the lower chamber was filled with 10% FBS RPMI. After 24 hours, invading cells were counted and the average number ± SD of cells are reported as fold change respect to control (SCR) considered as 100. All the experiments have been performed at least in triplicate ± standard deviation (SD) and p-value < 0.05 was considered as significant (Student’s t-test). **Suppl. Fig. 5.** a. LOX IMVI SEN cells were transiently transfected with miR-4443, miR-4488 or with the SCR for 72 hours and then cells have been stained with an antibody PE mouse anti-nestin for 45 minutes to perform flow cytometry analyses. Non-stained cells were used as negative control. Black arrows indicate the peaks relative to cells transfected with miR-4443 or miR-4488 mimics (left graphs). Quantitative analyses were calculated as “fold change” (± SD.) compared to SCR considered as 1 (right graph). Data were analyzed using CytExpert version 2.2 software. b. LOX IMVI SEN cells, transfected as described above, have been harvested and lysed to extract total proteins that have been subjected to Western blot analyses with the indicated antibodies. Tubulin levels have been estimated for the protein equal loading. c. LOX IMVI SEN and RES cells were subjected to Western blot analyses as described above. Tubulin levels have been estimated for the protein equal loading. d. LOX IMVI SEN and RES cells were subjected to flow cytometry analyses as described above. Non-stained cells were used as negative control. Black arrow indicates the peaks relative to RES cells (left graph). Quantitative analyses were calculated as “fold change” (± S.D.) compared to SEN cells considered as 1 (right panel). Data were analyzed using CytExpert version 2.2 software. All the experiments have been performed at least in triplicate ± standard deviation (SD) and p-value < 0.05 was considered as significant (Student’s t-test). **Suppl. Fig. 6.** a. A375 SEN, RES and DRES cells (double resistant to BRAFi and MEKi) were harvested and lysed to extract total proteins to perform Western blot analyses with the indicated antibodies. Tubulin levels have been estimated for the protein equal loading. b. Representative images by confocal microscopy of phalloidin, nestin and DAPI immunostaining of A375 SEN and DRES cells that were cultured into 8-well μ-slides for 48 hours. Magnification 63x. **Suppl. Fig. 7.** a. The online software GEPIA 2.0 (http://gepia2.cancer-pku.cn/#index) was interrogated to unveil nestin prognostic value based on Skin Cutaneous Melanoma (SKCM) data from The Cancer Genome Atlas (TCGA). Patients have been stratified according to the mutational status and the Kaplan Meier (KM) curves represent the data relative to the BRAF-wild type subtypes (n=164). b. Nestin levels (NES) were evaluated by immunohistochemistry (IHC) in 14 melanoma patients’ biopsies collected before starting MAPKi therapy together with the expression levels of miR-4443 and miR-4488 (miRNAs) quantified by qRT-PCR. Data have been used to plot KM curves to assess the predictive value of NES+miRNAs for therapy response (internal validation cohort). High and low levels were assessed by considering positive and negative z-scores, respectively. The hazard ratio, Cox models and the log-rank p values were evaluated to plot KM curves.

**Additional file 2: Suppl. Table 1. **It contains a) the clinical data relative to the 14 melanoma patients whose biopsies were collected before starting MAPKi therapy and b) the expression levels of nestin by immunohistochemistry (IHC) in these biopsies that have been determined counting positive cells in at least ten fields and expressed as percentage of positivity.

## Data Availability

The datasets used and/or analysed during the current study are available from the corresponding author on reasonable request.

## Abbreviations

MAPKi: BRAF and MEK inhibitors
miRNAs: MicroRNAs
LNPs: Lipid nanoparticles
RES: Drug resistant melanoma cells
DRES: Double resistant cells
SKCM: Skin Cutaneous Melanoma
TCGA: The Cancer Genome Atlas
RTCA: Real Time Cell Analysis
IHC: Immunohistochemistry
SCR: Scrambled
GEO: Gene Expression Omnibus
TIMP2: Tissue inhibitors of metalloproteinase 2
NES: Nestin
LNA: Locked nucleic acid
