# Supplementary material for: Upregulated expression of miR-4443 and miR-4488 in drug resistant melanomas promotes migratory and invasive phenotypes through downregulation of intermediate filament nestin
Source: J Exp Clin Cancer Res. 2023 Nov 27;42:317. doi: 10.1186/s13046-023-02878-9 (PMC10680267; doi:10.1186/s13046-023-02878-9)
Supplement: Supplementary file 1 — Additional file 1: Suppl. Fig. 1. a. A375 sensitive cells (SEN) and their resistant counterparts (RES) were plated in a 96-wells plate. The number of viable cells was measured by quantification of the cellular ATP present at different time points (from 24 to 96 hours). Results were calculated as fold change (± SD) relative to the time point of 24 hours. b, c. To perform migration assays, LOX IMVI SEN and RES cells or A375 SEN and DRES cells (double resistant to BRAFi and MEKi) were seeded in serum-free media into the upper chamber of a transwell, whereas the lower chamber was filled with 10% FBS RPMI. After 8 hours, cells remaining on the top side of the membrane were counted and the average number ± SD of cells are reported as fold change respect to control considered as 100. d, e. To perform invasion assays, LOX IMVI SEN and RES cells or A375 SEN and DRES cells were seeded into the upper chamber of a transwell coated with Matrigel, whereas the lower chamber was filled with 10% FBS RPMI. After 24 hours, invading cells were counted and the average number ± SD of cells are reported as fold change respect to control considered as 100. Three independent experiments were performed; each experiment was performed at least in quadruplicate. All the experiments have been performed at least in triplicate ± standard deviation (SD) and p-value < 0.05 was considered as significant (Student’s t-test). Suppl. Fig. 2. a-d. xCELLigence RTCA technology was used to measure cell migration and invasion of LOX IMVI SEN and RES cells or A375 SEN and DRES cells (double resistant to BRAFi and MEKi). To evaluate migration (a, c), cells were seeded in serum-free medium on filters in the upper chamber, whereas the lower chamber was filled with 10% FBS RPMI. Cell migration was monitored for 8 hours. To evaluate invasion (b, d), cells were seeded in serum-free medium on filters coated with Matrigel in the upper chamber, whereas the lower chamber was filled with 10% FBS RPMI. Cell invasion was m [file 13046_2023_2878_MOESM1_ESM.pdf]

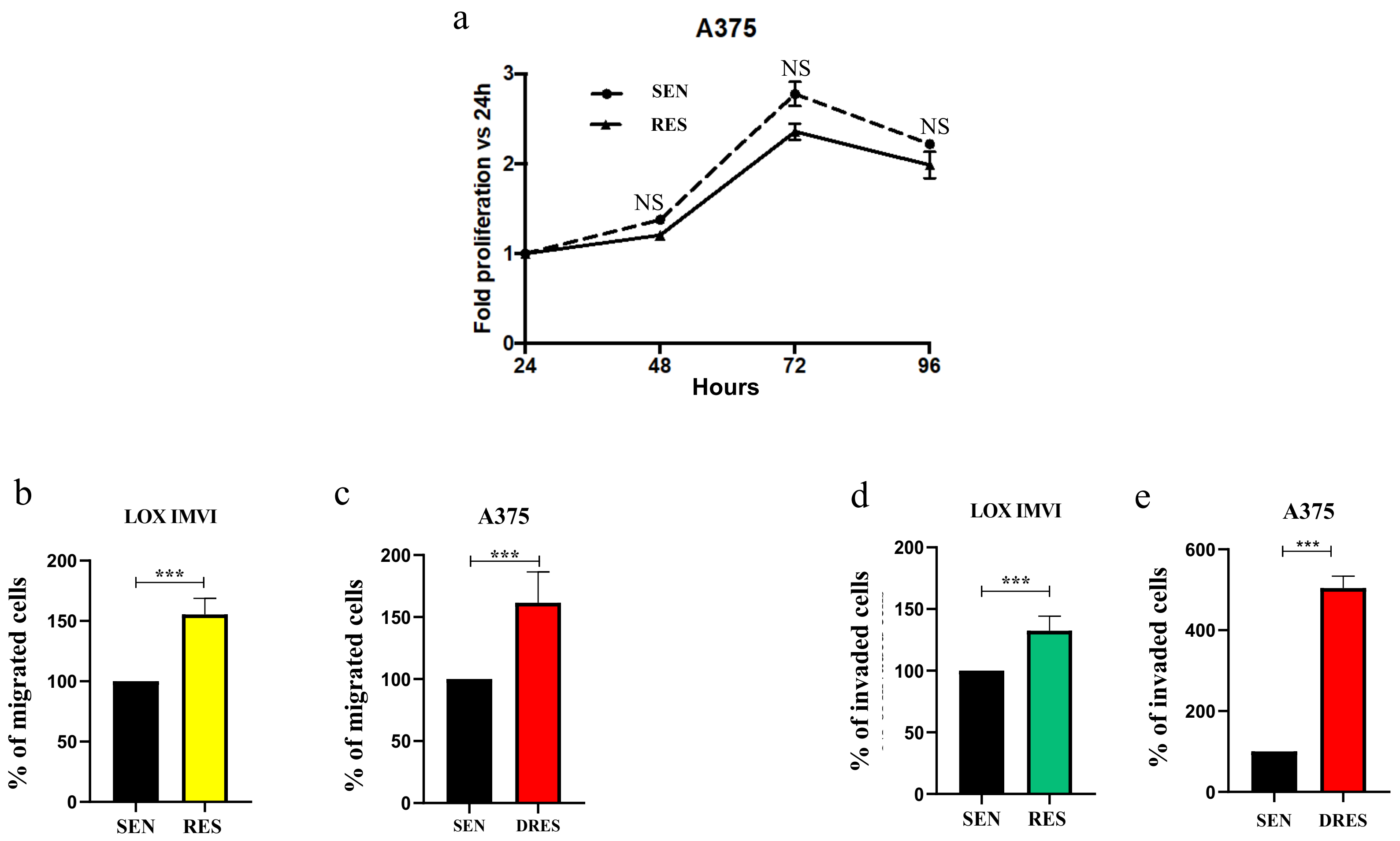

Suppl. Figure 1

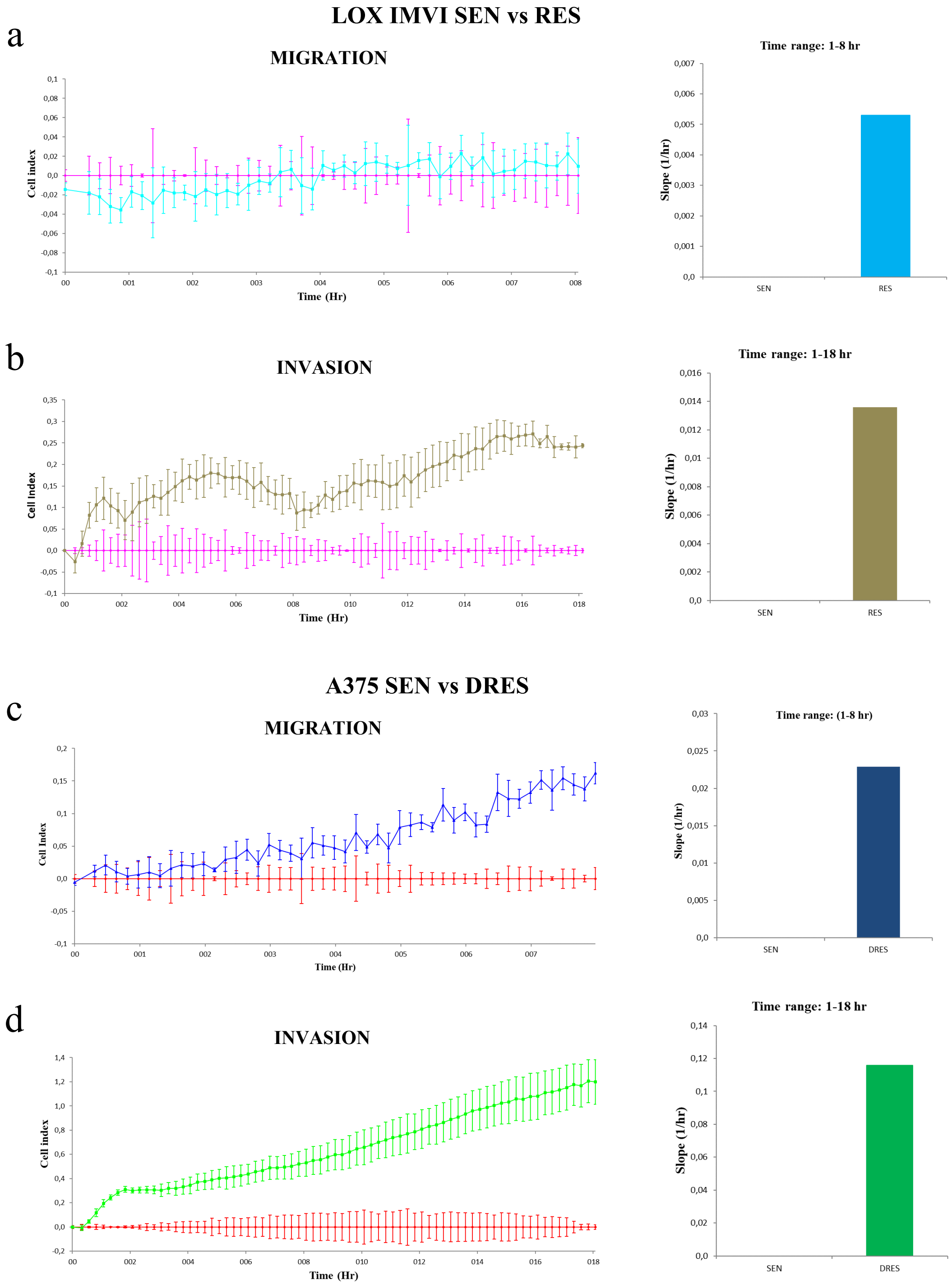

Suppl. Figure 2

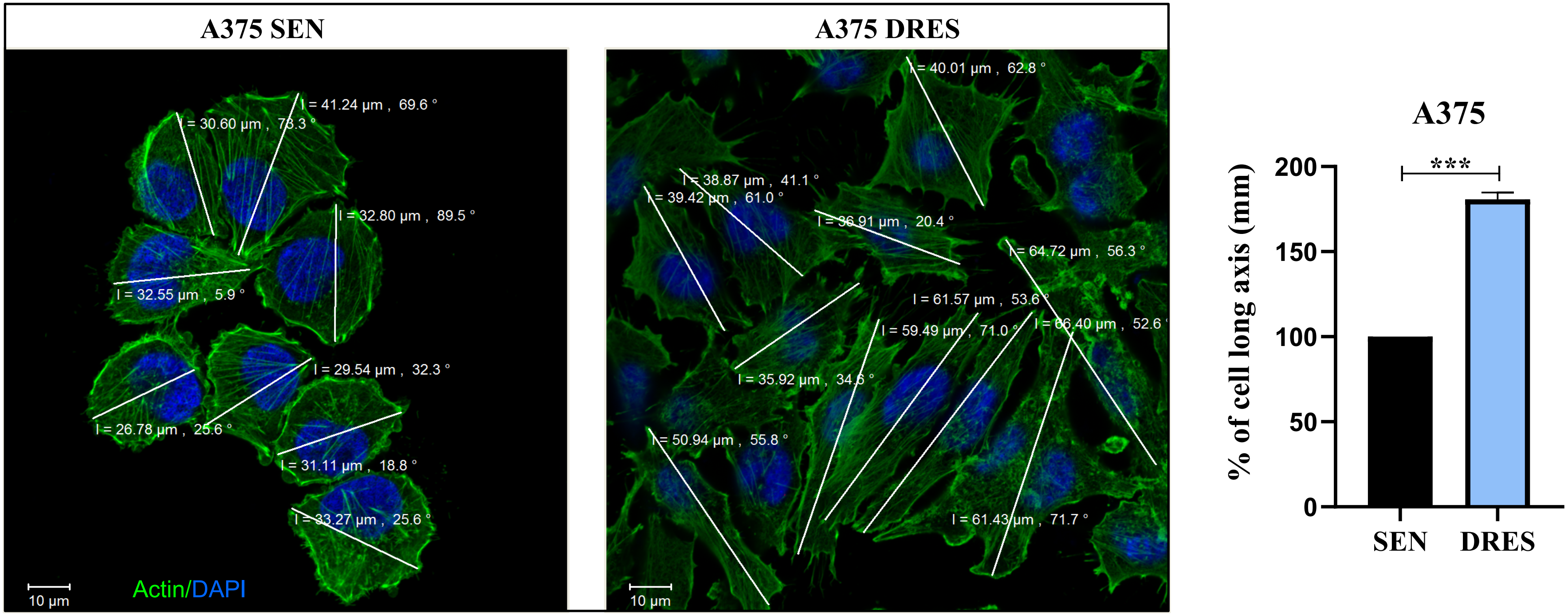

Suppl. Figure 3

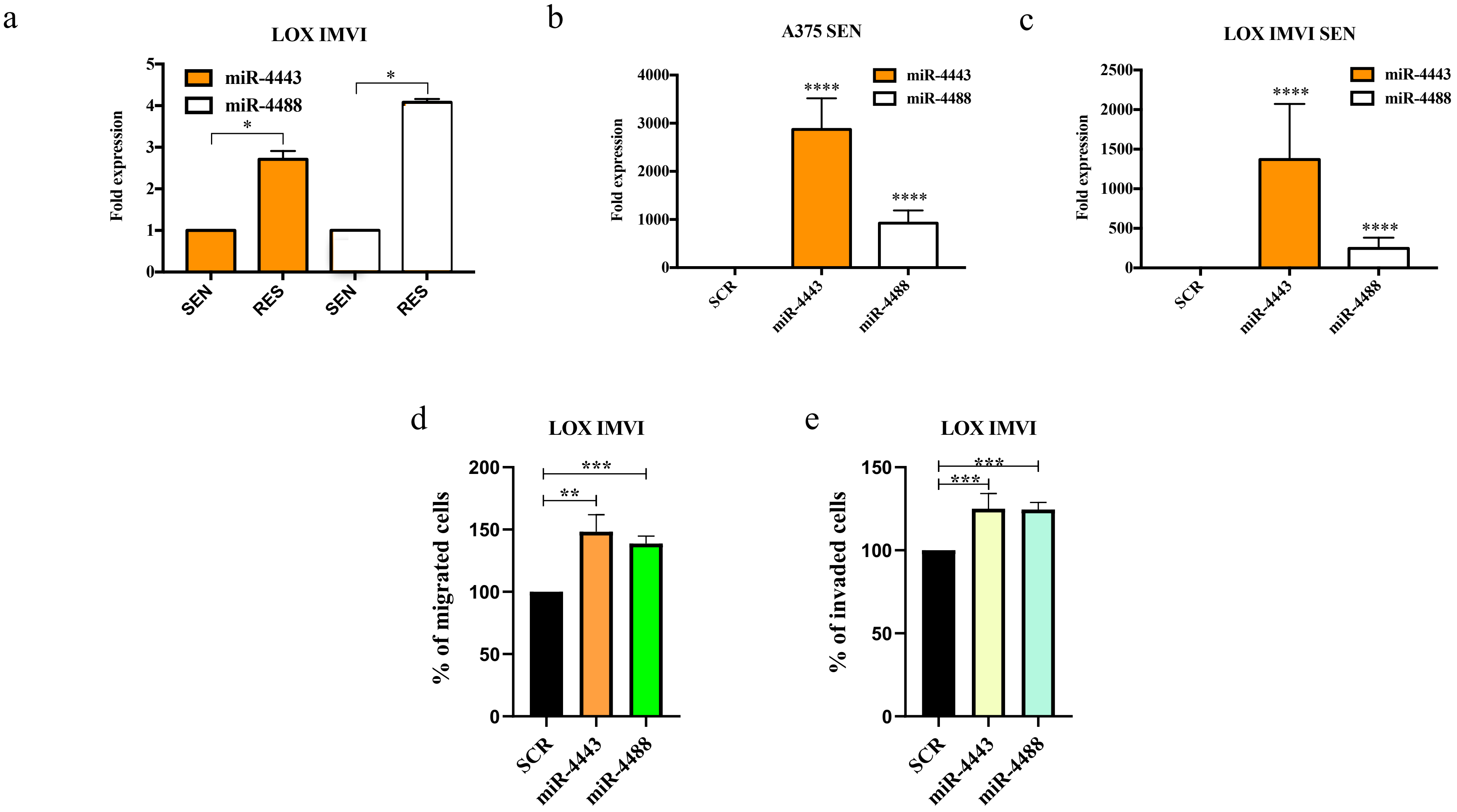

Suppl. Figure 4

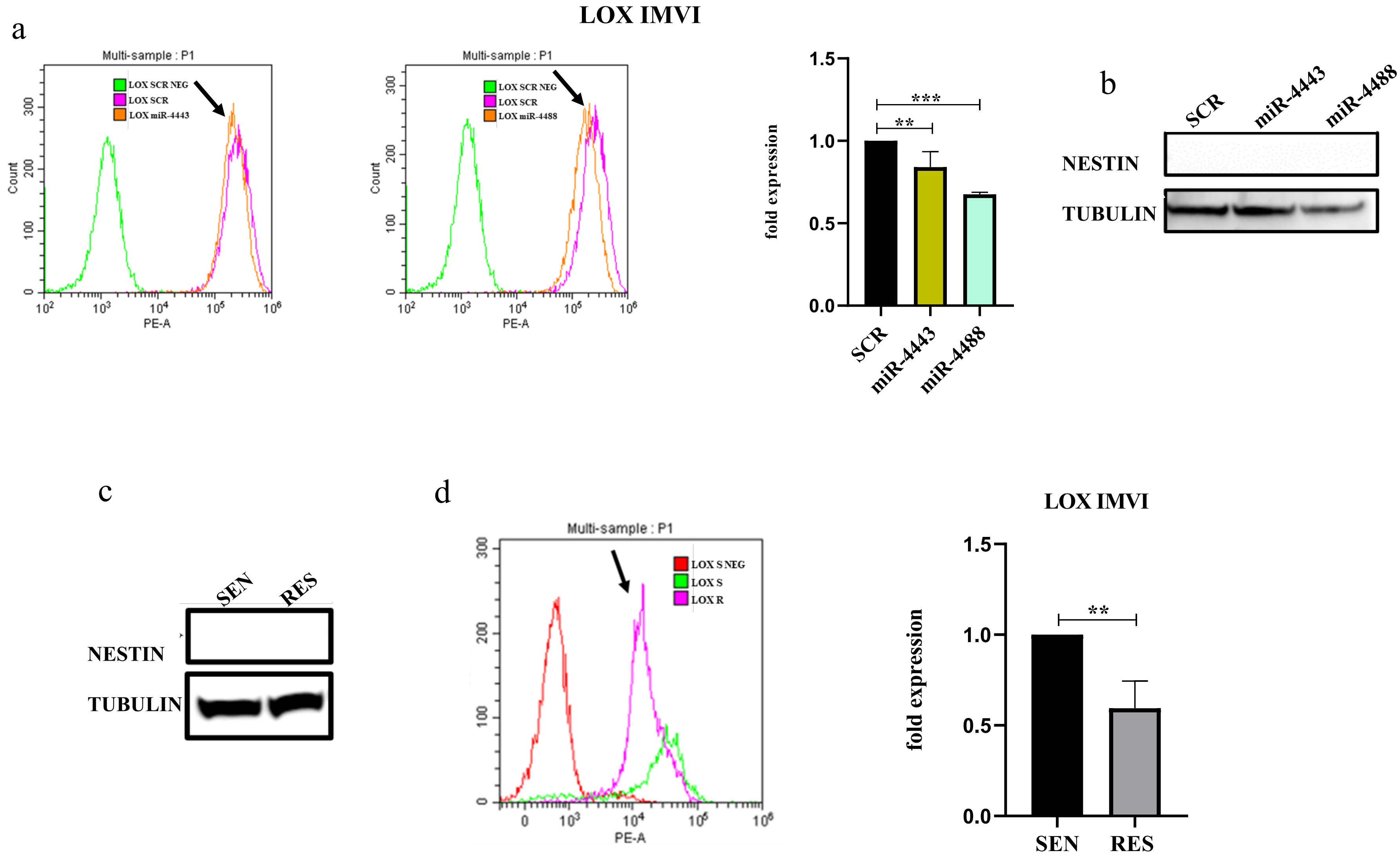

Suppl. Figure 5

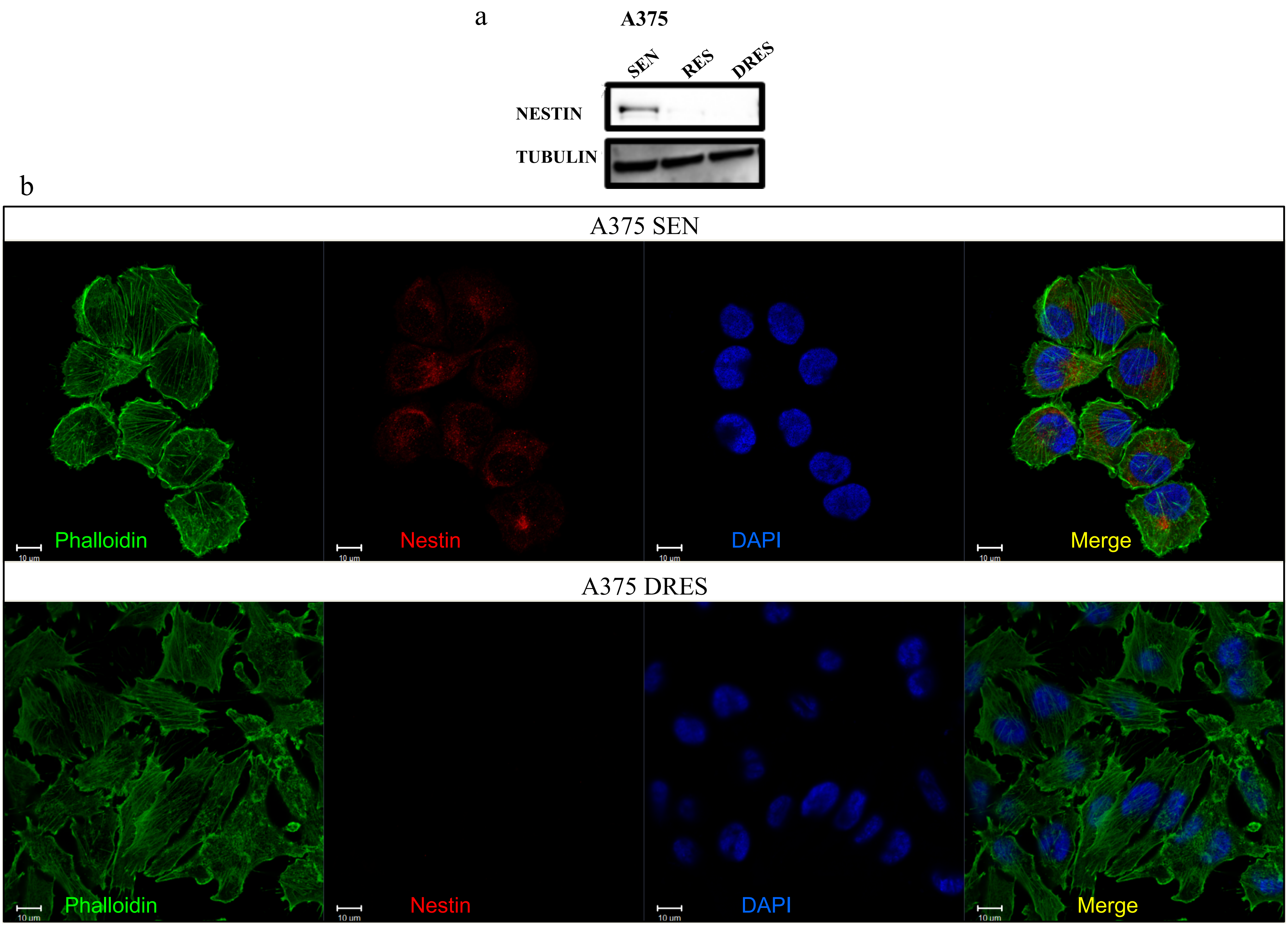

Suppl. Figure 6

BRAF-wt SKCM

a

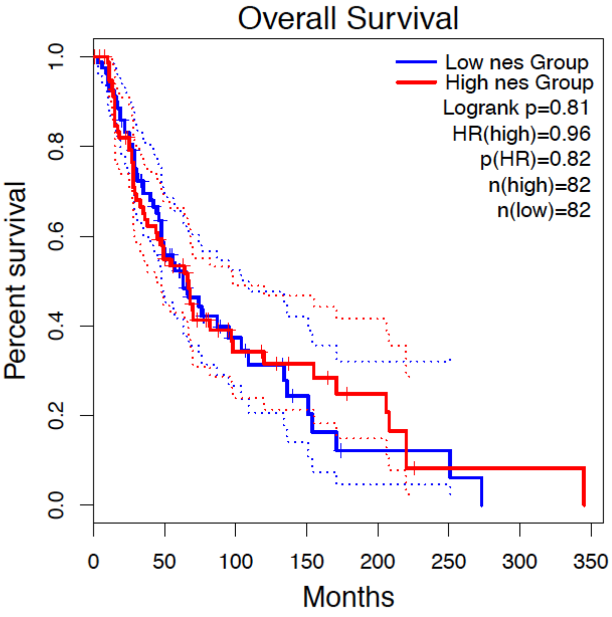

b

Internal validation cohort by IHC (n. patients=14)

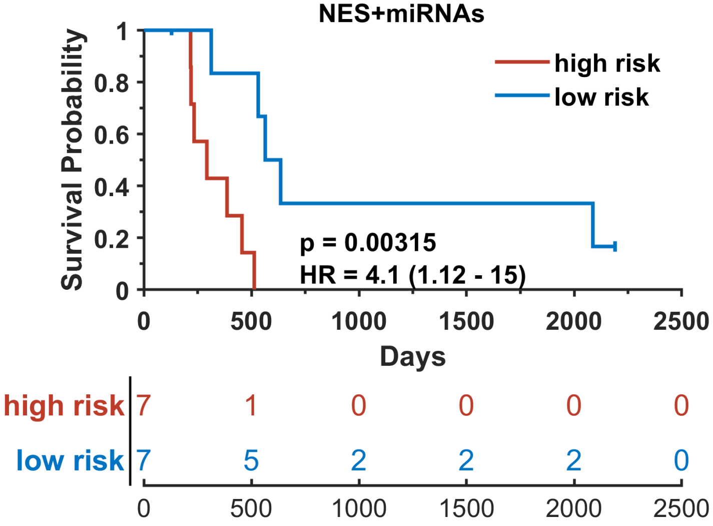

Suppl. Figure 7
